# Relationship of *MicroRNA-146a* rs2910164 SNP and tobacco consumption with the susceptibility of digestive system cancer

**DOI:** 10.1007/s12672-025-04193-5

**Published:** 2026-01-06

**Authors:** Jian Wang, Hao Qiu, Shuchen Chen, Ting Dai

**Affiliations:** 1 Department of Cardiothoracic Surgery, The Affiliated Yixing Hospital of Jiangsu University, Yixing, Jiangsu Province China; 2 Department of Laboratory Medicine, School of Medicine, Jiangsu University, Zhenjiang, Jiangsu Province China; 3 Department of Thoracic Surgery, Fujian Medical University Union Hospital, Fuzhou, 350001 Fujian Province China; 4 Department of Pharmacy, The Affiliated Yixing Hospital of Jiangsu University, Yixing, 214200 Jiangsu Province China

**Keywords:** MicroRNA-146a, Polymorphism, Tobacco, Digestive system cancer

## Abstract

Many studies reported that *microRNA* (*miR*)*-146a* rs2910164 could influence the risk of digestive system cancer (DSC). The purpose of this meta-analysis was to assess a possible effectof rs2910164 C to G variant in *miR-146a* gene and the smoking status with risk of DSC. The EMBASE, PubMed and CBM databases were searched to retrieve eligible papers up to January 20, 2025. Finally, 11 independent case-control studies involving 17,484 subjects were included. The association of rs2910164 polymorphism and tobacco using with DSC susceptibility was evaluated. In this pooled-analysis, we found significant difference in DSC risk between tobacco users and non-tobacco users who carried rs2910164 CC/CG genotypes (OR = 1.39, 95% CI = 1.05–1.84). In addition, there was significant difference between rs2910164 polymorphism (dominant model) and DSC susceptibility in both non-tobacco (OR = 1.19, 95% CI = 1.06–1.33) and tobacco users (OR = 1.13, 95%CI = 1.01–1.26). When stratified analyses were conducted by different type of DSC, we found significant difference in esophageal squamous cell carcinoma (ESCC) risk between tobacco users and non-tobacco users who carried rs2910164 CC/CG genotypes (OR = 2.33, 95% CI = 1.55–3.50). We also found significant difference between rs2910164 (dominant model) and gastric carcinoma (GC) susceptibility in both non-tobacco (OR = 1.27, 95% CI = 1.02–1.58). In summary, the current pooled-analysis highlights that tobacco consumption significantly increases the DSC development in rs2910164 CC/CG genotypes.

## Introduction

Digestive system cancer (DSC) is a major public health problem especially in Asians, which influences patients’ survival seriously. DSCs, such as pancreatic cancer (PC), gallbladder cancer (GBC), gastric carcinoma (GC), hepatocellular cancer (HCC), esophageal carcinoma (EC), oral carcinoma (OC), and colorectal carcinoma (CRC), are commonly diagnosed malignancies and lead to serious cancer-related death worldwide. Notwithstanding the risk factor of DSCs was not well explained, more and more investigations suggested that DSCs might be triggered by interactions of genetic components and environmental factors. Since single nucleotide polymorphism (SNP) is a common genetic variant, it might be involved in the development and progression of DSCs along with multiple environmental factors.

MicroRNA (miR), in eukaryotes, is composed of about 22 single strand nucleotide acids. Accumulating evidences indicated that miRs could regulate the expression of their target genes. A number of studies demonstrated that miR could play a vital role in regulating multiple biological functions via controlling target genes [10, 12, 27, 39–41, 54]. Tobacco consumption has been considered as an important risk factor for DSC. There was growing evidence that miRs could participate ininflammatory, immune, apoptosis, proliferation, migration, and invasion [6, 24, 32, 42, 44, 45, 49, 59]. *MiR-146a* involved inaprocess of posttranscriptional regulatory. Several studies demonstrated that *miR-146a* is implicated in the development of DSCs [5, 14]. A recent investigation suggested that *miR-146a* could facilitate oncogenesis of CRC and influence tumor microenvironment [8]. In addition, Li et al. reported that *miR-146a* combining with cigarette smoke extract could reduce the level of inflammatory factorsin lung adenocarcinoma cells [26]. Thus, *miR-146a* might combine with tobacco consumption and then affected the susceptibility of DSCs.

SNPs influence the miRs’ biological function and stability, lead to disorderin regulating target gene. M*iR-146a* rs2910164, a C→G variant, might alter the chance of survival in CRC patients via influencing the level of cyclooxygenase-2 and apoptosis process [57]. Jiang et al. found that rs2910164 CC/CG genotypes and tobacco using could significantly increase the risk of CRC [20]. In addition, arecent investigation also found that tobacco using and *miR-146a* rs2910164 CC/CG genotypes conferred a risk to esophageal squamous cell carcinoma (ESCC) and GC [29]. However, some case-control studies suggested that tobacco using and *miR-146a* rs2910164 CC/CG genotypes did not alter the risk of DSCs. On the contrary, Xia et al. reported that tobacco using and *miR-146a* rs2910164 CC/CG genotypes could decrease the risk of GC [50]. A few pooled-analyses indicated that rs2910164 SNP could affect the susceptibility of DSC [28, 33, 51–53]. Nowadays, several publications focused on the association of rs2910164 SNP and tobacco using with the development of DSCs. However, none pooled-analysis was performed. Since the eligible information was pooled and the random error could be reduced in meta-analysis, the confidence level of investigation might be promoted. In this study, we used a meta-analysis to evaluate a potential correlation of rs2910164 genotype and tobacco using with DSC susceptibility.

## Materials and methods

### Searching

The potential literatures were searched in CBM, Pubmed, and Embase databases by harnessing the following strategy: (rs2910164 OR microRNA-146a2 OR miR-146a2) AND (cancer OR carcinoma) AND (polymorphism OR SNP) AND (smoking OR tobacco OR cigarette) (the last searching data was January 20, 2025). To obtain data as much as possible, we retrieved the bibliographies of eligible studies. There was no language restriction in the current study.

### Inclusion criteria

Publications meeting the major included criteria were recruited in this meta-analysis: (a) case-control study; (b) considering *miR-146a2* rs2910164SNP and tobacco using with DSCsusceptibility; and (c) data could be extracted from publications to assess the strength of these associations by using odds ratio (OR) with 95% confidence interval (CI). In this study, details were not restricted for selection: (a) the years and number of tobacco using, and (b) type of tobacco.

### Information extraction

According to a standard protocol, the corresponding information was extracted by two authors (H. Qiu and J. Wang) independently. We obtained the following data: the first author’s surname, tobacco using status, publication years, country, genotyping method, ethnicities, type of DSC, study design (hospital-based or population-based study), and the number of rs2910164 genotypes for different tobacco using status. If there was conflicting measurement, another author (T. Dai) would take part in a discussion and make a vote. Finally, consistent opinions had been reached.

### StatisticalAnalysis

In this study, the pooled-analysis was conducted by using Stata12.0 software, (Stata Corp., College Station, Texas). *P* value (two-sided) ≥ 0.05 were defined as no statistical significance. The calculated ORs with 95%CIs were harnessed to observe the strength of the relationships. By using the *miR-146a2* rs2910164 genotypes in controls and Pearson’s *χ*^2^ test, the *P* value of Hardy–Weinberg equilibrium (HWE) was calculated for each included case-control study. The heterogeneity among the studies included in this pooled-analysis was measured using *I*^2^-test and *Q* statistics-test. When no significance for heterogeneity was found (*P* ≥ 0.1 and *I*^*2*^ < 50%), a fixed-effects model (Mantel–Haenszel) could be harnessed to obtain a measurement of this SNP and tobacco using with the susceptibility of DSC [34]. Otherwise, random-effects model (DerSimonian and Laird method) was employed to calculate a relationship of this SNP and tobacco using with DSC [11, 17]. Sensitivity analysis was carried out to evaluate the stability of our observations by omitting each individual study in turn, and calculating ORs with 95%CIs of the remainders. The possible bias of publication was evaluated by using Egger’s test and Begg’s test. And *P* < 0.05 was used as statistical significance for bias. The Newcastle-Ottawa Quality Assessment Scale (NOQAS) was harnessed to assess quality score [2, 4, 23, 25, 35, 47].

## Results

### Overall characteristics

**Fig. 1:**
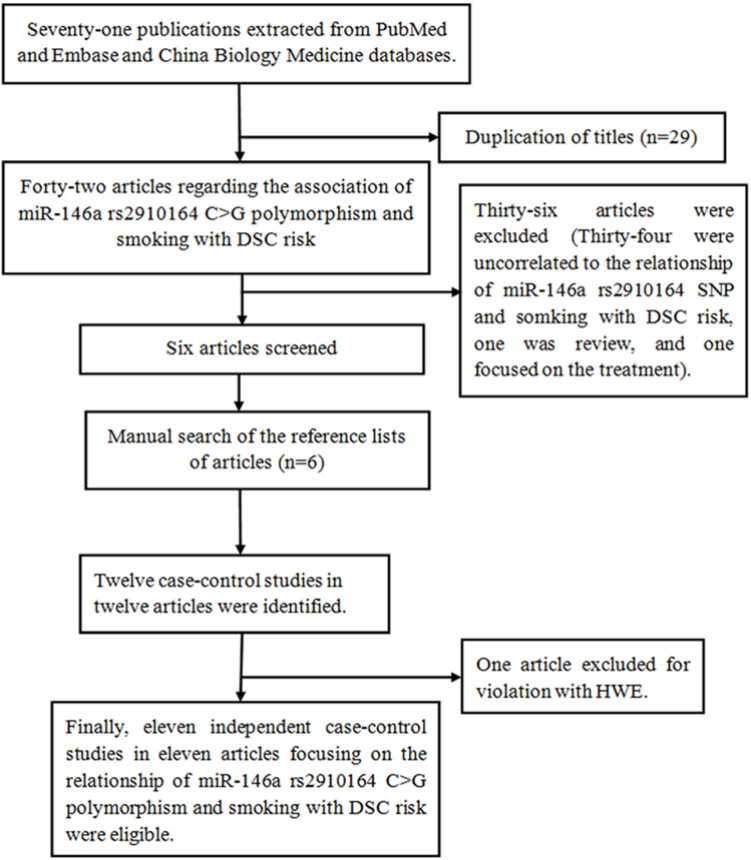
Flow diagram of the meta–analysis

A total of 71 publications were retrieved from Embase, PubMed and China Biology Medicine (CBM) databases. When a detailed filtrate made, 60 publications were ineligible. The selecting process is listed in Fig. 1. Finally, 11 investigations involving 7051 cases of DSC and 10,433 controls were included [1, 7, 9, 13, 16, 20, 21, 50, 55, 56]. Of them, publication years ranged from 2010 to 2025 and the participants’ number ranged between 525 and 2740. In brief, there were five studies focusing on GC [1, 7, 21, 50, 55], two studies on ESCC [16, 20], two studies on CRC [13, 20] and 2 studies on HCC [9, 56]. Additionally, alleligible studies were performed on Asian populations. Table 1 presents characteristics of the included publications. The distribution of rs2910164 genotypes is summarized in Table 2.

**Table 1:** Characteristics of the studies in meta-analysis

Author	Year	Sample size	Cancer type	Country	Year	Study design	Quality
Zeng et al.	2010	304/304	GC	China	2010	HB	8
Guo et al.	2010	444/468	ESCC	China	2010	HB	7
Ahn et al.	2013	461/447	GC	Korea	2013	HB	7
Chu et al.	2014	188/337	HCC	China	2014	HB	8
Xia et al.	2016	1125/1196	GC	China	2016	PB	8
Chen et al.	2018	1063/1677	GC	China	2018	HB	7
Lin et al.	2018	490/1476	GC	China	2018	HB	7
Gao et al.	2018	560/780	CRC	China	2018	HB	7
Zhang et al.	2020	584/923	HCC	China	2020	HB	7
Jiang et al.	2021	1003/1303	CRC	China	2021	HB	7
Liu et al.	2022	829/1522	ESCC	China	2022	HB	7

*GC* Gastric cancer;

*CRC* Colorectal cancer;

*HCC* Hepatocellular cancer;

*ESCC* Esophageal squamous cell carcinoma;

*HB* Hospital-based

*PB* Population-based

**Table 2:** Distribution of miR-146a rs2910164 C > G genotypes and smoking status

The association between the miR-146a rs2910164 C>G polymorphism and tobacco use on cancer risk.						
Author		Year	Case-No. of user CC	Control-No. of user CC	Case-No. of non-user CC	Control-No. of non-user CC
Between tobacco users and non-tobacco users among individuals homozygous for the major allele (CC)	Zeng *et al.*	2010	21	21	68	95
	Guo *et al.*	2010	7	20	13	22
	Ahn* et al.*	2013	33	47	24	117
	Chu* et al.*	2014	34	47	50	94
	Xia *et al.*	2016	148	206	249	214
	Chen *et al.*	2018	99	138	228	506
	Lin *et al.*	2018	69	174	113	409
	Gao *et al.*	2018	67	102	78	118
	Zhang *et al.*	2020	79	130	115	229
	Jiang *et al.*	2021	94	111	274	405
	Liu *et al.*	2022	160	159	134	421

Author		Year	Case-No. of user CG+GG	Control-No. of user CG+GG	Case-No. of non-user CG+GG	Control-No. of non-user CG+GG
Between tobacco users and non- tobacco users who are minor allele carriers (CG+GG)	Zeng *et al.*	2010	52	34	163	151
	Guo *et al.*	2010	289	227	135	199
	Ahn* et al.*	2013	68	87	74	196
	Chu* et al.*	2014	46	65	58	131
	Xia *et al.*	2016	291	380	437	396
	Chen *et al.*	2018	188	215	526	815
	Lin *et al.*	2018	110	249	194	640
	Gao *et al.*	2018	193	277	222	283
	Zhang *et al.*	2020	128	197	253	365
	Jiang *et al.*	2021	158	154	454	630
	Liu *et al.*	2022	267	260	244	675

Author		Year	Case-No. of non-user CG+GG	Control-No. of non-user CG+GG	Case-No. of non-user CC	Control-No. of non-user CC
Between non- tobacco users homozygous for the major allele and non- tobacco users who are minor allele carriers	Zeng *et al.*	2010	163	151	68	95
	Guo *et al.*	2010	135	199	13	22
	Ahn* et al.*	2013	74	196	24	117
	Chu* et al.*	2014	58	131	50	94
	Xia *et al.*	2016	437	396	249	214
	Chen *et al.*	2018	526	815	228	506
	Lin *et al.*	2018	194	640	113	409
	Gao *et al.*	2018	222	283	78	118
	Zhang *et al.*	2020	253	365	115	229
	Jiang *et al.*	2021	454	630	274	405
	Liu *et al.*	2022	244	675	134	421

Author		Year	Case-No. of user CG+GG	Control-No. of user CG+GG	Case-No. of user CC	Control-No. of user CC
Between tobacco users homozygous for the major allele and tobacco users who are minor allele carriers	Zeng *et al.*	2010	52	34	21	21
	Guo *et al.*	2010	289	227	7	20
	Ahn* et al.*	2013	68	87	33	47
	Chu* et al.*	2014	46	65	34	47
	Xia *et al.*	2016	291	380	148	206
	Chen *et al.*	2018	188	215	99	138
	Lin *et al.*	2018	110	249	69	174
	Gao *et al.*	2018	193	277	67	102
	Zhang *et al.*	2020	128	197	79	130
	Jiang *et al.*	2021	158	154	94	111
	Liu *et al.*	2022	267	260	160	159

### Quantitative synthesis

**Fig. 2:**
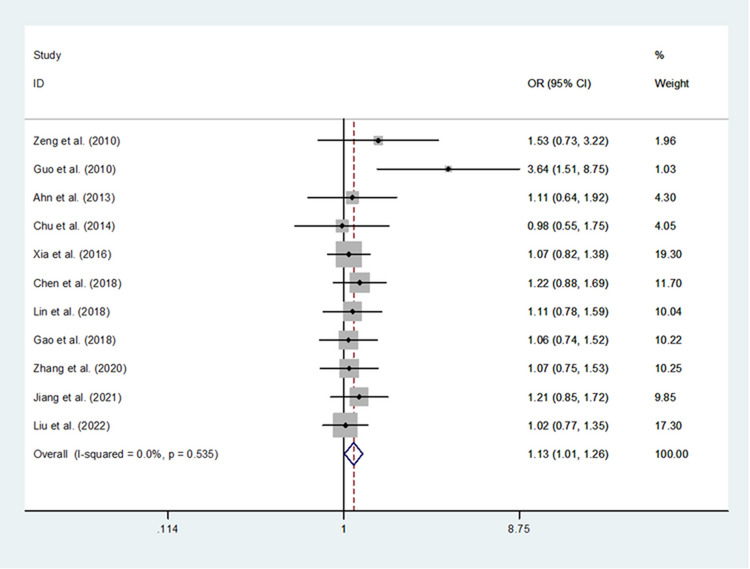
Meta-analysis of between tobacco users homozygous for the major allele and tobacco users who are minor allele carriers (fixed–effects model)

Table 3 presents all main results. In this pooled-analysis, we identified no difference in DSC susceptibility between tobacco users and non-tobacco users who carried rs2910164 CC genotype (OR = 0.137, 95% CI = 0.98–1.92, *P* = 0.063). However, we found significant difference in DSC risk between tobacco users and non-tobacco users who carried rs2910164 CC/CG genotypes (OR = 1.39, 95% CI = 1.05–1.84). In addition, there was significant difference between rs2910164 SNP (dominant model) and DSC susceptibility in both non-tobacco (OR = 1.19, 95% CI = 1.06–1.33) and tobacco users (Fig. 2, OR = 1.13, 95%CI = 1.01–1.26). In view of these findings, tobacco consumption could increase DSC susceptibility compared to non-tobacco consumption, regarding rs2910164 CC/CG genotypes.

**Table 3:** Results of the meta-analysis

No. of studies		Between tobacco users and non-tobacco users among individuals homozygous for the major allele (CC)				Between tobacco users and non- tobacco users who are minor allele carriers (CG + GG)			
		OR (95% CI)	*P*	*I*^*2*^	*P* (Q-test)	OR (95% CI)	*P*	*I*^*2*^	*P* (Q-test)
Total	11	1.37 (0.98–1.92)	0.063	87.0%	< 0.001	**1.39 (1.05–1.84)**	**0.021**	90.1%	< 0.001
Cancer type									
GC	5	1.42 (0.83–2.42)	0.204	89.2%	< 0.001	1.29 (0.87–1.92)	0.197	89.1%	< 0.001
ESCC	2	1.49 (0.29–7.65)	0.630	88.0%	0.004	**2.33 (1.55–3.50)**	** < 0.001**	80.5%	0.023
HCC	2	1.25 (0.93–1.69)	0.144	0.0%	0.730	1.18 (0.70–1.97)	0.538	71.4%	0.062
CRC	2	1.15 (0.90–1.48)	0.272	0.0%	0.389	1.12 (0.74–1.79)	0.618	85.0%	0.010
Sample sizes									
< 1,000	4	1.53 (0.82–2.84)	0.180	67.6%	0.026	**1.79 (1.48–2.17)**	** < 0.001**	0.0%	0.642
≥ 1,000	7	1.32 (0.87–1.98)	0.188	91.0%	< 0.001	1.24 (0.86–1.80)	0.243	93.8%	< 0.001
Quality scores									
≥ 8	3	1.00 (0.54–1.86)	0.997	78.5%	0.010	1.13 (0.62–2.05)	0.700	86.2%	0.001
< 8	8	**1.55 (1.11–2.16)**	**0.009**	82.3%	< 0.001	**1.50 (1.13–1.99)**	**0.006**	89.1%	< 0.001

No. of studies		Between non- tobacco users homozygous for the major allele and non- tobacco users who are minor allele carriers				Between tobacco users homozygous for the major allele and tobacco users who are minor allele carriers			
		OR (95% CI)	*P*	*I*^*2*^	*P* (Q-test)	OR (95% CI)	*P*	*I*^*2*^	*P* (Q-test)
Total	11	**1.19 (1.06–1.33)**	**0.003**	40.6%	0.078	**1.13 (1.01–1.26)**	**0.041**	0.0%	0.535
Cancer type									
GC	5	**1.27 (1.02–1.58)**	**0.032**	65.6%	0.020	1.14 (0.97–1.34)	0.123	0.0%	0.903
ESCC	2	1.14 (0.90–1.43)	0.275	0.0%	0.978	1.80 (0.52–6.20)	0.355	86.4%	0.007
HCC	2	1.11 (0.68–1.81)	0.676	70.6%	0.065	1.04 (0.77–1.42)	0.784	0.0%	0.798
CRC	2	1.10 (0.93–1.30)	0.293	0.0%	0.585	1.14 (0.88–1.46)	0.325	0.0%	0.605
Sample sizes									
< 1000	4	1.29 (0.90–1.84)	0.162	50.3%	0.110	1.45 (0.88–2.38)	0.149	55.1%	0.083
≥ 1000	7	**1.17 (1.07–1.28)**	**0.001**	40.8%	0.119	1.10 (0.97–1.24)	0.127	0.0%	0.983
Quality scores									
≥ 8	3	1.06 (0.77–1.46)	0.728	61.2%	0.076	1.09 (0.87–1.36)	0.466	0.0%	0.619
< 8	8	1.23 (1.12–1.35)	** < 0.001**	20.3%	0.269	**1.14 (1.01–1.30)**	**0.044**	11.4%	0.342

*GC* Gastric cancer;

*CRC* Colorectal cancer;

*HCC* Hepatocellular cancer;

*ESCC* Esophageal squamous cell carcinoma

When stratified analyses were conducted by different type of DSC, we found significant difference in ESCC risk between tobacco users and non-tobacco users who carried rs2910164 CC/CG genotypes (OR = 2.33, 95% CI = 1.55–3.50). We also found significant difference between rs2910164 SNP (dominant model) and GC susceptibility in non-tobacco using participants (OR = 1.27, 95% CI = 1.02–1.58).

### Heterogeneity

Significant heterogeneity was found between rs2910164 SNP and DSC susceptibility in CC, CC/CG genotypes, and non-tobacco consumption subgroup ( *P* < 0.001, *P* < 0.001 and *P* = 0.078). However, we found related low heterogeneity between tobacco users with CC genotype and tobacco users with CG/GG genotype and DSC susceptibility (*P* = 0.535). We conducted subgroup analysis to assess the potential reason for significant heterogeneity. This meta-analysis indicated that GC, ESCC, HCC, sample size ≥ 1,000 participants and NOQAS ≥ 8 subgroups might lead to major heterogeneity of this pooled-analysis.

### Sensitivity analysis

**Fig. 3:**
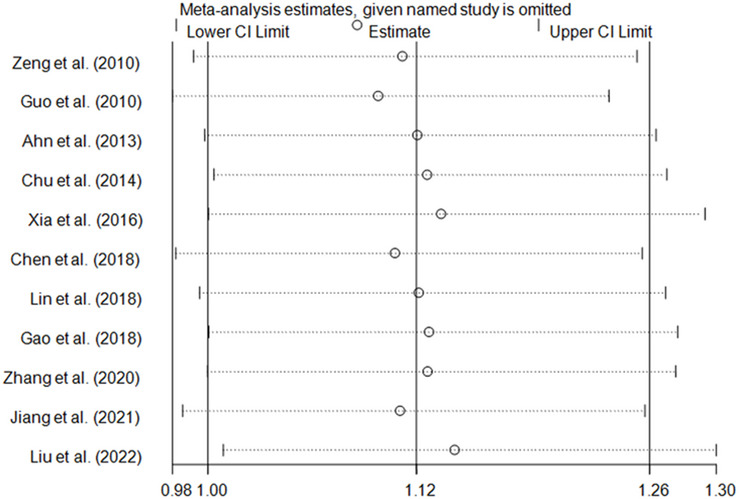
Sensitivity analysis of meta–analysis (between tobacco users homozygous for the major allele and tobacco users who are minor allele carriers, fixed–effects model)

In this pooled-analysis, we conducted sensitivity analysis to assess the reliability of our findings. We found that omitting any independent study could not significantly change the results of meta-analysis (Fig. 3).

### Publication bias

**Fig. 4:**
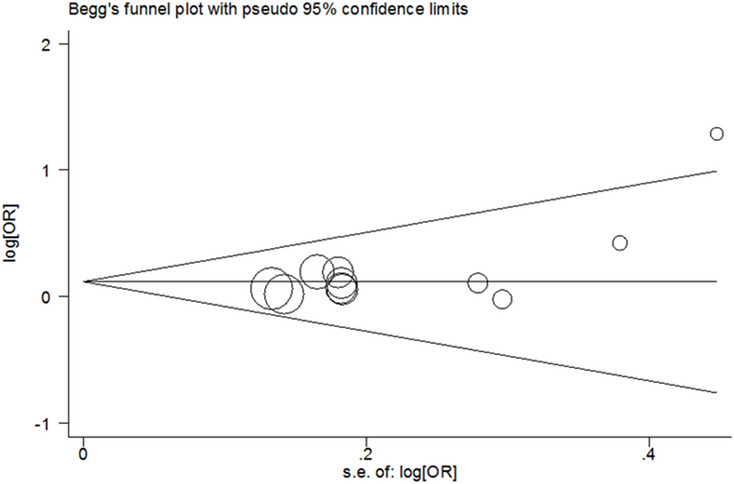
Begg’s funnel plot of meta–analysis (between tobacco users homozygous for the major allele and tobacco users who are minor allele carriers, fixed–effects model)

**Fig. 5:**
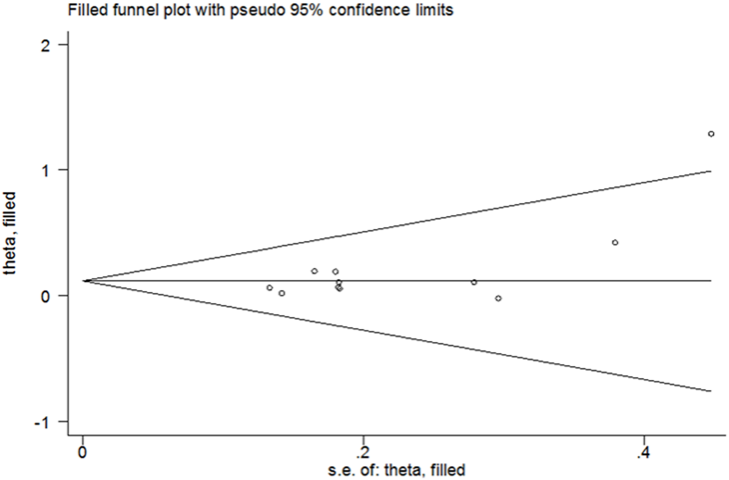
Results of nonparametric “trim-and-fill” method (between tobacco users homozygous for the major allele and tobacco users who are minor allele carriers, fixed–effects model)

Bias analysis for publication indicated that significant bias had been happened between rs2910164 SNP (dominant model) and DSC susceptibility in tobacco users (Begg’s Test: *P* = 0.213, Egger’s test: *P* = 0.029, Fig. 4). In other comparison, we did not find any publication bias (data not shown). In this study, we performed nonparametric “trim-and-fill” method to adjust the potential bias [3, 15, 38, 43, 46, 48, 58]. The results indicated no trimming performed and data unchanged (Fig. 5). Findings were calculated as following: OR = 1.13, CI = 1.01–1.26, *P* = 0.041, indicating the reliability of our findings.

## Discussion

In this investigation, a systematic review and pooled-analysis was conducted to assess a potential relationship of *miR-146a2* rs2910164 SNP and tobacco using with DSC susceptibility. We observed the following findings: (a)The current pooled-analysis suggested that tobacco using could significantly increase the DSC development in rs2910164 CC/CG genotypes. Additionally, this study indicated that a potential correlation of rs2910164CC/CG genotypes and tobacco using with DSC susceptibility in ESCC subgroup, and (b) In tobacco using subjects, rs2910164 CC/CG genotype also could significantly increase the DSC development.

The current pooled-analysis indicated that rs2910164 CC/CG genotypes increased a susceptibility of DSC among non-tobacco users. These observations were similar to the previous meta-analysis for the association between rs2910164 SNP and DSC development [30]. Additionally, among tobacco users, rs2910164 CC/CG genotypes increased a susceptibility of DSC. These conflicting findings in the present pooled-analysis could be interpretated by the included sample sizes. Admittedly, in non-tobacco users, there was a relationship of DSC susceptibility with rs2910164 CC/CG genotypes. The potential link between rs2910164 SNP and DSC development was: (a) *miR-146a* linked with the invasion and migration of cancer [31]; (b) the expression of *miR-146a* was associated with immunosuppressive tumor microenvironment and drug resistant [22]; and (c) G allele was correlated with the level of *miR-146a* [18, 19, 36]. Nadi et al. reported that a significantly decreased expression of miR-146a in cigarette smoke exposure subjects [37]. Li et al. reported that *miR-146a* combining with cigarette smoke extract could reduce the level of inflammatory factors in lung adenocarcinoma cells [26]. Here, we might speculate that the rs2910164 SNP and cigarette consumption could affect *miR-146a* level and lead to DSC susceptibility.

In the present study, significant heterogeneity was found in several comparison. We assessed the source of heterogeneityby subgroup analysis. The GC, ESCC, larger sample size (≥ 1,000 subjects), and high quality score (≥ 8) subgroups might lead to significant heterogeneity.

Some merits could be addressed in this pooled-analysis. Firstly, the current meta-analysis indicated a possible association of rs2910164 SNP and tobacco using with DSC risk. Secondly, the sample sizes included in this meta-analysis were large. The observations could be less bias. Finally, in the present meta-analysis, we found that tobacco using could significantly increase the DSC development in rs2910164 CC/CG genotypes.

Some limitations also should be acknowledged. Firstly, all included investigations were designed in Asian populations, and other populations was not considered. Thus, these observations might be only adapted to Asians. Secondly, we only focused on the association of rs2910164 SNP and tobacco using with DSC susceptibility. The potential confounders (e.g., age, sex, alcohol use, H. pylori infection for gastric cancer, hepatitis status for HCC) and issues of population structure were not adequately considered. The findings should be interpreted more cautiously. Thirdly, in certain comparison, publication bias had been identified. Fourthly, the definition of smoking was inconsistent across the included studies. Finally, in the current pooled-analysis, we only considered the association of rs2910164 SNP and tobacco using with DSC susceptibility. And the potential relationship of another vital *miR-*SNPs and tobacco using with DSC susceptibility also should been taken into account. In the future, more studies with prospective cohorts, Mendelian randomization, and functional assays should be carried out to better address causality.

In summary, the current pooled-analysis highlights that tobacco using significantly increases the DSC development in rs2910164 CC/CG genotypes. In the future, the potential relationship of rs2910164 SNP and other habits with DSC susceptibility also should been taken into account.

## Data Availability

Data is provided within the manuscript or supplementary information files.
